# The Prognostic Value of the CD8^+^PD-1^+^/CD4^+^PD-1^+^ (PERLS) Ratio for Leukemic Transformation in MDS

**DOI:** 10.3390/hematolrep18020029

**Published:** 2026-04-15

**Authors:** Panagiotis Panagiotidis, Emmanuel Karavanis, Konstantinos Neanidis, Eleftherios Panteris, Maria Moysidou

**Affiliations:** 1 Oncology/Hematology Clinic, 424 General Military Hospital (424 GMHT), 56429 Thessaloniki, Greece; 2 Biomedical Sciences Unit, Faculty of Health Sciences, Metropolitan College, Campus of Thessaloniki, 54626 Thessaloniki, Greece; epanteris@mitropolitiko.edu.gr; 3 Neonatology/Neonatal Intensive Care Unit (NICU), University General Hospital of Heraklion, University of Crete, 71003 Heraklion, Greece

**Keywords:** myelodysplastic syndromes, acute myeloid leukemia, PD-1, CD8^+^ T cells, CD57^+^, CD8^+^PD-1^+^/CD4^+^PD-1^+^ (PERLS)

## Abstract

**Background/Objectives:** Myelodysplastic syndromes (MDS) are associated with a significant risk of progression to acute myeloid leukemia (AML), affecting approximately 30% of patients. In high-risk MDS, leukemic transformation may occur within a short time frame, highlighting the need for early and reliable biomarkers of disease progression. Increasing evidence suggests that immune dysregulation and cytotoxic T-cell dysfunction contribute to disease evolution. This study aimed to evaluate PD-1 and CD57 expressions on CD8^+^ T cells and to investigate the CD8^+^PD-1^+^/CD4^+^PD-1^+^ ratio (PERLS) as a potential immunological marker predictive of leukemic transformation. **Methods:** Thirty-one patients with MDS were prospectively followed over a 12-month period. At baseline, patients underwent routine clinical and laboratory evaluation, including multiparameter flow cytometric assessment of bone marrow blasts. An extended immunophenotypic analysis of bone marrow samples was performed at study entry to assess PD-1 and CD57 expression on CD8^+^ T cells. Cytogenetic and molecular analyses were conducted when clinical findings suggested disease progression. Patients who developed signs of progression were re-evaluated approximately one month later, during the progression phase, to assess dynamic immunological changes. **Results:** Of the thirty-one patients included, eighteen progressed to AML, whereas thirteen remained clinically stable. Patients who progressed demonstrated a significant increase in PD-1 and CD57 expression on CD8^+^ T cells compared with stable patients. Moreover, a markedly higher CD8^+^PD-1^+^/CD4^+^PD-1^+^ (PERLS) ratio was observed in patients who subsequently developed AML, particularly during the progression phase. **Conclusions:** Dynamic immunophenotypic monitoring reveals that increased PD-1 on CD8^+^ T cells and an elevated PERLS ratio are associated with imminent leukemic transformation in MDS. These findings support the incorporation of immune-based biomarkers, particularly the CD8^+^PD-1^+^/CD4^+^PD-1^+^ ratio, into routine risk assessment to enable earlier identification of disease progression and timely therapeutic intervention.

## 1. Introduction

Myelodysplastic syndromes (MDS) are a heterogeneous group of clonal hematopoietic stem cell disorders characterized by ineffective hematopoiesis, peripheral blood cytopenias, bone marrow dysplasia and an increased risk of transformation to acute myeloid leukemia (AML) [1,2]. Approximately one-third of MDS patients eventually progress to AML [3]. Both MDS and AML have an increased prevalence in the aging population and may demonstrate resistance to treatment [4].

The progression of patients with myelodysplastic syndromes (MDS) to acute myeloid leukemia (AML) is a complex biological process involving genetic, epigenetic, molecular, immunophenotypic and immunologic alterations [5,6,7,8]. This transformation is clinically defined by an increase in bone marrow blasts to ≥20% of total nucleated cells and is commonly referred to as secondary AML evolving from MDS [3]. During leukemic transformation, hematopoietic stem or progenitor cells acquire genetic abnormalities that give rise to malignant leukemic stem cells (LSCs), which sustain clonal expansion of immature blasts [3]. Leukemic stem cells (LSCs) are typically characterized by an immature immunophenotypic profile, including expression of markers such as CD34^+^, CD117 (c-kit), and TIM-3 [9]. Advances in cytogenetics and molecular profiling now integrate classic karyotype analysis with targeted gene panels to refine diagnosis, risk stratification, and monitoring of MDS progression toward AML [10,11]. In the present study, we focused on somatic mutations in FLT3, NPM1, TP53 and RUNX1, which have been implicated as key molecular drivers of leukemic transformation in MDS patients [11].

Flow cytometry immunophenotyping has been established as a valuable complementary tool in evaluating myelodysplastic syndromes, contributing to the detection of phenotypic abnormalities and aiding in diagnostic and prognostic assessment, particularly in specialized centers or when morphology and cytogenetics are indeterminate [12,13]. Leukemic progression in MDS is strongly influenced by dysregulation of immune checkpoint pathways [14]. Among these, the programmed cell death protein 1 (PD-1) plays a critical role in facilitating immune escape [15]. PD-1, expressed on exhausted CD8^+^ T cells, inhibits effector functions including cytokine production (e.g., IFN-γ and TNF-α), cytotoxic degranulation, and proliferation [16]. Furthermore, its ligand, PD-L1, is frequently upregulated on CD34^+^ leukemic blasts and contributes to immune suppression by acting on T cells and regulatory T cells [17,18]. Dysregulation of the PD-1/PD-L1 axis promotes leukemogenesis by supporting clonal expansion, inducing apoptosis of normal hematopoietic cells, and suppressing anti-leukemic immune responses [14]. Another important marker expressed on CD8^+^ T cells is CD57, which has traditionally been associated with senescence and exhaustion. According to recent guidelines, CD57^+^ CD8^+^ T cells are more accurately classified within the Short-Lived Effector Cell (SLEC) compartment, representing highly differentiated, activated CD8^+^ T cells with cytotoxic activity that undergo apoptosis during the transition from an activated to a resting state [19,20]. Elevated CD57^+^ CD8^+^ T cells in the bone marrow of AML patients are associated with poor anti-leukemic immunity, predict treatment failure, correlate with worse overall survival, and persist in non-responders following chemotherapy [21].

The expression levels of T-cell exhaustion markers, including PD-1 and CD57, not only reflect their immunoregulatory roles but have also been linked to clinical outcomes in MDS and AML, serving as predictors of adverse prognosis, particularly in higher-risk subgroups [14,16,21,22]. Elevated PD-1 expression on CD8^+^ T cells correlate with inferior overall and relapse-free survival, particularly following hypomethylating agent therapy. Likewise, expansion of CD8^+^CD57^+^ senescent T-cell subsets has been associated with diminished cytotoxic capacity and poor outcomes in advanced disease stages [20,21]. Together, these findings suggest that PD-1 and CD57 may serve as immunologic biomarkers of leukemic transformation and prognosis. Nonetheless, their prognostic significance remains partly controversial, as studies evaluating bulk peripheral-blood mononuclear cells have produced inconsistent results, likely due to mixed lymphocyte profiles and underlying clonal heterogeneity [23]. Notably, alterations in immune cell subsets between lower- and higher-risk patients, along with correlations with clinical outcomes and treatment response, have been reported, reflecting that immune dysfunction is a clinically meaningful factor influencing disease progression and prognosis [24]. It is now recognized that CD8^+^PD-1^+^ T cells in MDS are a functionally heterogeneous population, including stem-like progenitor exhausted cells capable of self-renewal and differentiation into effector T cells [25]. Consequently, PD-1 expression does not necessarily indicate irreversible immune dysfunction, highlighting the dynamic nature of T-cell exhaustion and its potential impact on disease progression and therapeutic responses [16,25]. Thus, in MDS, stem-like PD-1^+^ CD8^+^ T-cell subsets have been shown to influence the evolving immune microenvironment, modulate anti-leukemic responses, and represent potential targets for immunotherapeutic interventions [25,26].

While immunological dysregulation has been identified in both MDS and AML patients, there is a lack of longitudinal studies characterizing specific immune population dynamics during the transition from MDS to AML. This study highlights the utility of immunophenotypic profiling to characterize T-cell exhaustion through established markers such as PD-1 and CD57 on CD8^+^ T cells and introduces the CD8^+^PD-1^+^/CD4^+^PD-1^+^ ratio (PERLS) as a novel biomarker of early immune dysregulation during progression from MDS to AML. The PERLS ratio was initially described in patients with non-small cell lung cancer as a prognostic indicator of response to PD-(L)1 blockade [27], but its relevance in myeloid neoplasms remains unexplored.

Therefore, the aim of this study is to determine whether longitudinal assessment of the CD8^+^PD-1^+^/CD4^+^PD-1^+^ (PERLS) ratio can serve as a predictive marker of immune exhaustion and leukemic evolution in patients with MDS.

## 2. Materials and Methods

### 2.1. Study Design

A total of 31 patients newly diagnosed with myelodysplastic syndrome (MDS) were prospectively enrolled in the study between 2023 and 2025 at the Hematology Clinic of the 424 General Military Training Hospital of Northern Greece (424 GSNE). Patients were monitored over a 12-month follow-up period to evaluate disease progression from MDS to acute myeloid leukemia (AML), with some patients followed for up to 18 months.

### 2.2. Ethics and Exclusion Criteria

This study received ethical approval from the Directorate of Health Services, Hellenic Army General Staff (Protocol No. F.330/1/773—Serial No. 74, dated 2 January 2024), and was conducted in accordance with the Declaration of Helsinki and relevant military research guidelines. Informed consent was obtained from all participating patients. Patients were excluded if they had concurrent hematologic or solid malignancies, incomplete clinical data, or had not provided informed consent. All participants underwent routine infection screening at enrollment, and only those without evidence of ongoing or prior infection were eligible (To be included in the study, patients were required to have had a diagnosis of MDS for at least six months prior to enrollment, ensuring adequate follow-up and monitoring. Formal enrollment, prospective follow-up, and all experimental procedures, including flow cytometry, were initiated only after ethics approval was obtained on 2 January 2024. No study-related data were collected, and no protocol-driven procedures were performed prior to ethics approval.

### 2.3. Patient Evaluation and Classification

For MDS patients, diagnosis was established according to the 2017 World Health Organization (WHO) classification criteria for MDS [28], supplemented by the Ogata scoring system [29] and IPSS-R score [30]. Patients were evaluated based on morphological assessment of blast percentage in bone marrow aspirate smears and biopsy specimens, along with immunophenotypic analysis. MDS patients were classified into subtypes according to WHO 2016/2017 criteria: MDS-SLD (Single Lineage Dysplasia), MDS-MLD (Multilineage Dysplasia), MDS-RS (Ring Sideroblasts), MDS-EB (Excess Blasts), and MDS-U (Unclassifiable). Most newly diagnosed patients had an Ogata score ≥3. Based on the available clinical history and review of the patients’ medical records, all cases were considered de novo MDS. Patients who did not exhibit features of leukemic transformation (*n* = 13; 9 men, 4 women) were classified as the MDS group (Time 0) and served as controls. All MDS patients were screened for the JAK2 V617F mutation using molecular analysis. Patients positive for JAK2 V617F were subsequently tested for BCR-ABL1 to exclude Philadelphia chromosome–positive myeloid neoplasms. At the same time, additional MDS patients were present in the hospital but were not included in the study either because they declined participation, did not fully meet the study inclusion criteria, or had missing measurements.

**Figure 1:**
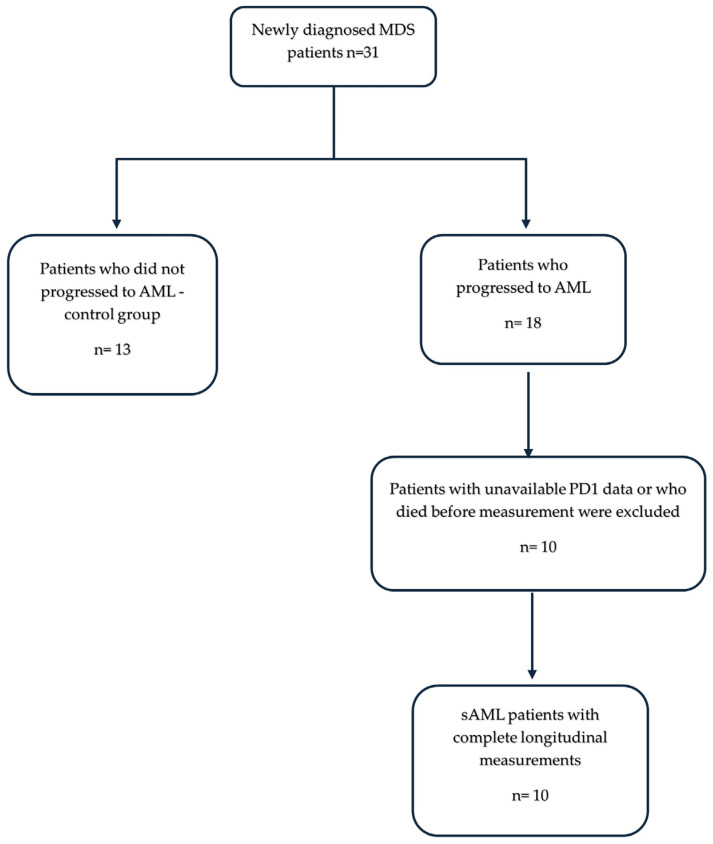
Flow diagram of secondary AML patient selection.

Patients who progressed to AML were included in the AML group (n = 18; 13 men, 5 women). AML was defined by ≥20% blasts in the bone marrow. Cases were classified according to FAB subtypes (M0–M7), based on morphologic features, while cytogenetic and molecular results (targeted gene panel including FLT3, NPM1, and RUNX1) were collected. All 18 patients who progressed to AML were included in the AML cohort. However, complete measurements across longitudinal time points were available for 10 patients and are presented in the figures, while the remaining 8 either died before study completion or had incomplete data, such as missing PD-1 measurements at the time of bone marrow blast assessment (Figure 1).

Bone marrow aspirates and biopsy specimens were collected and analyzed at three consecutive time points for blast percentage and morphological features: Time 0 corresponded to the initial assessment of MDS patients, at which point bone marrow samples were collected and blast counts were measured, and all related measurements were obtained. Time 1 corresponded to the onset of clinical symptoms indicative of disease progression, at which point bone marrow samples were collected for blast cell quantification. Based on the blast counts, patients were classified as either still having MDS (n = 7; <20% blasts) or exhibiting early leukemic progression (n = 3; >20% blasts). At this time point, treatment was adjusted as shown in Table 1. Time 2 corresponded to the formal diagnosis of secondary AML (sAML), approximately one month later, when complete cytogenetic and molecular results were available. Cytogenetic analysis and molecular profiling were performed at Time 1.

Patients with low-risk MDS received supportive care for anemia, including iron supplementation and transfusions [31] or a low dose of azaticidine (75 mg/m^2^ per day × 5 days). Patients with intermediate MDS and high-risk MDS received azacitidine [32]. Patients who progressed to secondary AML were treated according to standard induction regimens (7 + 3: cytarabine and anthracycline) [33]. A subset of older patients received an alternative regimen consisting of standard/high dose azacitidine (130–150 mg/m^2^ per day) [34]. Details of individual patient treatments are provided in Table 1 and Supplementary Table S1. 

### 2.4. Immunophenotypic Analyses by Flow Cytometry

Immunophenotypic analyses of bone marrow samples at all three time points were performed using a pentachrome flow cytometryantibody panel (Beckman Coulter, Brea, CA, USA) on a BC FC500 cytometer (Beckman Coulter, Brea, CA, USA), and data were analyzed with Kaluza C^®^ software version 1.2 (Beckman Coulter, Brea, CA, USA)., as detailed below.

Blast populations were initially identified on CD45 versus CD34 dot plots using PC7-conjugated anti-CD45 (J33) and ECD-conjugated anti-CD34 (581). Blasts were defined as CD45dim/CD34^+^ events and subsequently confirmed by sequential gating on HLA-DR (FITC) versus CD117 (PE) expression (Supplementary Figure S1). Percentages of blast populations were calculated relative to total nucleated cells excluding erythroid precursors Samples were classified according to blast percentage as MDS (<20% blasts) or secondary AML (>20% blasts). CD45 expression was used to evaluate white blood cell differentiation, whereas CD34, CD117 (c-kit), and HLA-DR were employed to characterize abnormal neoplastic blast populations. Additional immunophenotypic markers were assessed to classify AML subtypes (M0–M7) in accordance with established guidelines [35]. Myeloid and monocytic markers including PC5-conjugated anti-CD33(D3HL60.251), PC5-conjugated anti-CD13(Immu103.44), PC5-conjugated anti-CD14(RMO52), FITC-conjugated anti-CD15(80H5), ECD-conjugated anti-CD16(3G8), FITC-conjugated myeloperoxidase cytoplasmic MPO-LF(CLB-MPO-1), PE-conjugated anti-CD117(95C3), FITC-conjugated HLA-DR(Immu-357), PC5-conjugated anti-CD38(LS198-4-3), and PC5-conjugated anti-CD11b(Bear1) were used, along with aberrant lymphoid markers such as FITC-conjugated anti-CD2(39C1.5) and FITC-conjugated anti-CD7(8H8.1), to define lineage and maturation stage.

The percentages of CD3^+^CD8^+^PD-1^+^ and CD3^+^CD8^+^CD57^+^ T cells in bone marrow were quantified by flow cytometry to assess T-cell exhaustion and terminal differentiation. CD3^+^CD8^+^PD-1^+^ cells were measured using PE-conjugated anti-CD279(PD1.3), ECD-conjugated anti-CD3(UCHT1), and PC5-conjugated anti-CD8(B9.11), whereas CD3^+^CD8^+^CD57^+^ cells were measured using FITC-conjugated anti-CD57(NC1), ECD-conjugated anti-CD3(UCHT1), and PC5-conjugated anti-CD8(B9.11). CD3^+^CD4^+^PD-1^+^ was also measured using PE-conjugated anti-CD279(PD1.3), ECD-conjugated anti-CD3(UCHT1), and PC5-conjugated anti-CD4(13B8.2).

### 2.5. Statistical Analyses

Descriptive statistics for the percentages of CD34^+^ cells within the blast population, as well as the percentages of CD3^+^ PD-1^+^, CD8^+^ PD-1^+^, and CD8^+^ CD57^+^ lymphocyte subsets, are presented as mean ± standard deviation ($\bar{X}$ ± SD). Flow cytometry parameters were compared across three groups (MDS, progression, and sAML) using the Kruskal–Wallis test, followed by post hoc pairwise comparisons with Dunn’s test and Bonferroni correction (adjusted significance level α = 0.0167). A *p*-value of <0.05 was considered indicative of statistical significance. All analyses were performed using IBM SPSS Statistics, version 26.0 (Armonk, NY, USA: IBM Corp.).

## 3. Results

### 3.1. Patients’ Characteristics

A total of 31 patients with newly diagnosed myelodysplastic syndromes (MDS) were included in the study and followed prospectively for over 12 months. Of these, 18 patients (13 men, 5 women) progressed to secondary acute myeloid leukemia (AML) and constituted the sAML group, whereas 13 patients (9 men, 4 women) who did not show evidence of leukemic transformation served as the ΜDS group (Figure 1). Clinical, morphological, cytogenetic and molecular characteristics of the sAML patients (n = 10) included in this study are summarized in Table 1. The median age at diagnosis was 65 years (range 44–79). The distribution of MDS subtypes according to WHO 2017/2022 criteria is summarized in Table 1. Among the cohort, one patient was classified as MDS-U, three as MDS-SLD, three as MDS-EB-1, two as MDS-EB-2, and one as MDS-MLD. According to the Revised International Prognostic Scoring System (IPSS-R), five patients were low-risk, three intermediate, and two high-risk. All patients received low-dose azacitidine from the time of MDS diagnosis, except for one elderly patient who received supportive care. The corresponding data for the 13 MDS patients who did not progress to AML during the same study period are provided in Supplementary Table S1.

The distribution of AML patients according to the FAB classification is presented in Table 1. The AML subtypes among the ten patients were characterized as follows, M0 (n = 3), M1 (n = 2), M2 (n = 1), M4 (n = 3), and M5 (n = 1), based on morphological evaluation of bone marrow smears supported by immunophenotypic analyses with flow cytometry, according to the FAB classification. Among the ten AML patients analyzed by the targeted gene panel, no mutations were detected in 4 cases, whereas 6 patients carried at least one genetic alteration. The most frequent mutations involved NPM1 (n = 2), FLT3 (n = 3), and RUNX1 (n = 1). Cytogenetic analysis revealed that 4 out of 10 AML patients exhibited a normal karyotype, whereas 6 patients displayed abnormal cytogenetic findings, most commonly complex karyotypes or recurrent chromosomal aberrations, including −Y, +8, del(20q), and various translocations (e.g., 46,0,[−2–5del(7)(q?31q?33), −8, add(11)(p11.2), −12, +15, −17). At Time 1 of progression to secondary AML, all patients who increased the percentage of blast cells (n = 10) had their therapy changed. As seen in Table 1, six patients received standard induction chemotherapy (7 + 3: cytarabine and anthracycline), while the remaining four were switched to high-dose azacitidine according to the treating physician’s assessment and each patient’s clinical status.

### 3.2. Immunophenotypic Characterization of Blasts from MDS to AML

**Figure 2:**
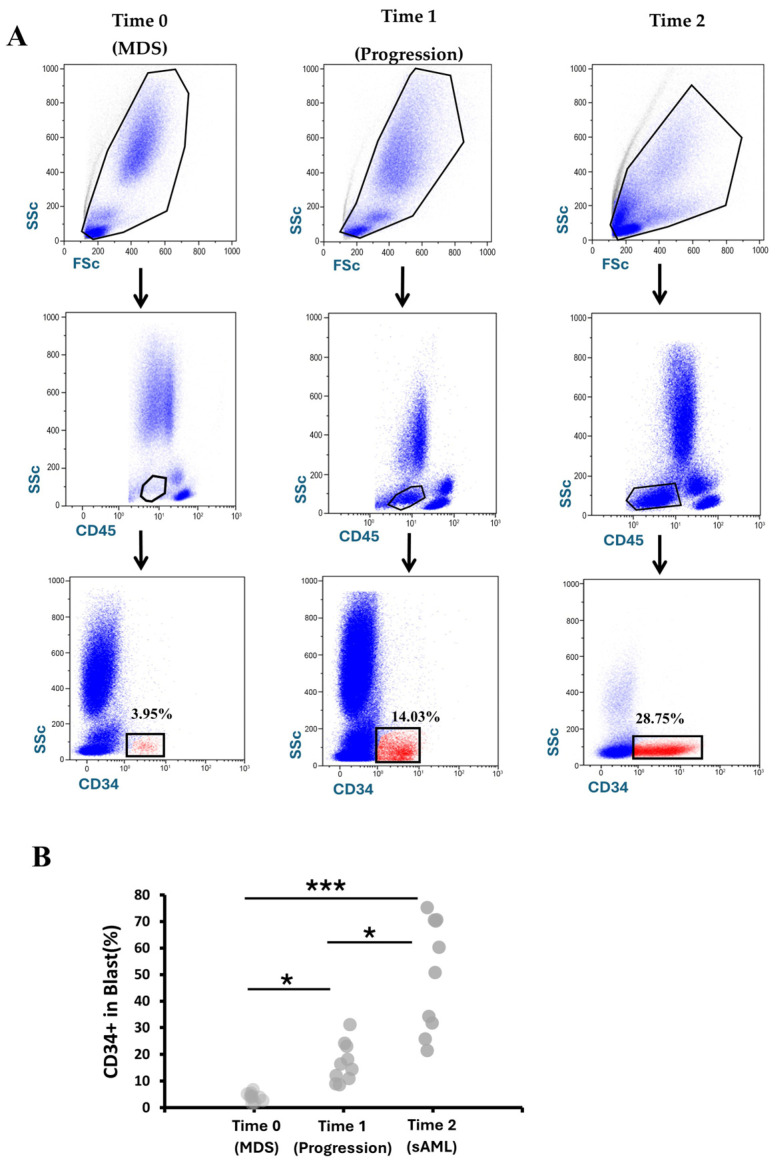
Flow cytometric assessment of blast population in bone marrow across study time points. (**A**) Representative flow cytometry analysis of CD34^+^ blast cells (red dots) gated within CD45^+^ cells in bone marrow samples from a single patient at three consecutive time points: Time 0 (MDS), Time 1 (disease progression), and Time 2 (sAML). Percentages shown are representative of the corresponding sample. (**B**) Percentages of CD34^+^ blasts at Time 0 (MDS, n = 10), Time 1 (progression, n = 10), and Time 2 (sAML, n = 10). Statistical significance is indicated in the figure (* *p* < 0.05; *** *p* < 0.001).

Flow cytometric immunophenotyping was performed on bone marrow aspirates obtained longitudinally from patients with myelodysplastic syndromes (MDS) who subsequently progressed to acute myeloid leukemia (AML). In a representative patient, CD34^+^ blasts within the CD45^+^ compartment increased from 3.93% at Time 0 (MDS) to 16.73% at Time 1 (progression) and 51.04% at Time 2 (sAML), reflecting expansion of the immature blast compartment; across the cohort, mean ± SD percentages were 3.93 ± 2.61% at Time 0, 16.73 ± 11.26% at Time 1, and 51.04 ± 26.89% at Time 2 (n = 10) (Figure 2A), with individual patient values summarized in Supplementary Table S2. Overall differences among groups were statistically significant by Kruskal–Wallis testing (H(2) = 15.47, *p* = 0.01), with post hoc pairwise comparisons confirming significant differences between all stages and demonstrating significantly higher CD34^+^ blast frequencies in sAML samples compared with both MDS and progression phase (Figure 2B). To further validate blast identification, a more stringent sequential gating strategy was applied. CD45^dim^/CD34^+^ events within the WBC compartment were re-gated on CD34^+^ cells and analyzed for HLA-DR and CD117 expression. This additional immunophenotypic refinement did not significantly modify blast frequencies (Supplementary Figure S1).

### 3.3. Immunophenotypic Profiling of CD8^+^ T Cells During MDS to AML Progression

**Figure 3:**
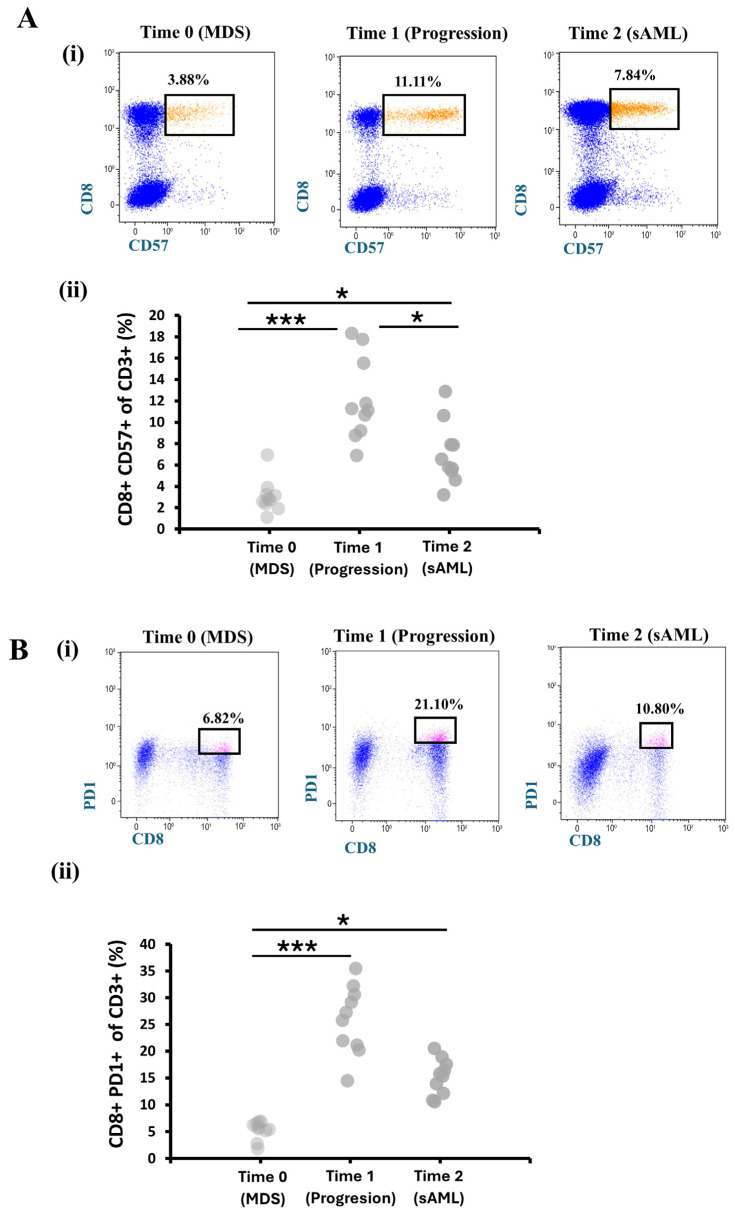
Immunophenotypic analyses of CD8^+^ T-cell subsets in bone marrow across study time points. (**A**) Representative percentages of CD8^+^CD57^+^ cells (orange dots) within CD3^+^ T cells in a bone marrow sample from a single patient at three consecutive time points different stages, Time 0 (MDS), Time 1 (disease progression), and Time 2 (sAML) (**i**), and corresponding percentages of the same cells from all patients (n = 10) at Time 0, Time 1, and Time 2 (**ii**). (**B**) Representative percentages of CD8^+^PD-1^+^ cells (pink dots) within CD3^+^ T cells in a bone marrow sample from a single patient at Time 0, Time 1, and Time 2 (**i**), and corresponding percentages from all patients (n = 10) at the same time points (**ii**). Statistical significance is indicated by * *p* < 0.05 and *** *p* < 0.01.

To evaluate the immunologic status of patients across study time points in MDS, we performed flow cytometry analysis to assess markers related to T-cell exhaustion or senescence of CD8^+^ T cells, such as PD-1 and CD57. Initially, we measured the expression of CD57 on CD8^+^ T cells to characterize the terminal differentiation of CD8^+^ T cells during disease progression. A representative flow cytometry plot from a single patient illustrates the frequency of CD8^+^CD57^+^ T cells within the CD3^+^ gate at Time 0 (MDS, 3.88%), Time 1 (progression, 11.11%), and Time 2 (sAML, 7.84%) (Figure 3A(i)). Quantitative analysis of the cohort revealed that the proportion of CD8^+^CD57^+^ T cells increased from 3.06% ± 2.92% at Time 0 (MDS) to 12.11% ± 5.73% at Time 1 (disease progression), followed by a decline to 7.03% ± 4.84% at Time 2 (sAML) (Figure 3A(ii)). Post hoc pairwise comparison showed a significant increase in CD8^+^ CD57^+^ T cells in the progression phase compared to Time 0 (MDS) (adjusted *p* = 0.001). Frequencies in sAML compared to MDS also reached statistical significance (adjusted *p* = 0.0167). These findings suggest an elevation of CD8^+^ CD57^+^ T cells during disease progression, with a subsequent decrease following AML transformation, coinciding with therapy initiation at Time 1.

Dysregulation and functional status were observed by measuring the CD8^+^ PD-1^+^ population within CD3^+^ T cells. Representative percentages of CD8^+^ PD-1^+^ T cells gated within the CD3^+^ population from a single patient accounted for 6.82% at Time 0 (MDS), 21.10% at Time 1 (progression) and 10.80% at Time 2 (sAML) (Figure 3B(i)). The proportion of CD8^+^PD1^+^ cells within the CD3^+^ T cells’ population was 5.29% ± 2.50% at Time 0 (MDS), which increased significantly to 25.79% ± 10.52% at Time 1 (progression), followed by a slight decrease to 15.16% ± 4.98% at Time 2 (sAML) (Figure 3B(ii)). Post hoc analysis pairwise comparison showed a significant increase in CD8^+^ PD-1^+^ cells in the progression phase compared to MDS (adjusted *p* = 0.000). Frequencies at Time 1 were higher compared to Time 2 but did not reach any statistical significance (*p* = 0.085).

### 3.4. Immunophenotypic Profiling of CD8^+^/CD4^+^ Ratio Within PD1^+^ CD3^+^ T Cells During MDS to AML Progression

**Figure 4:**
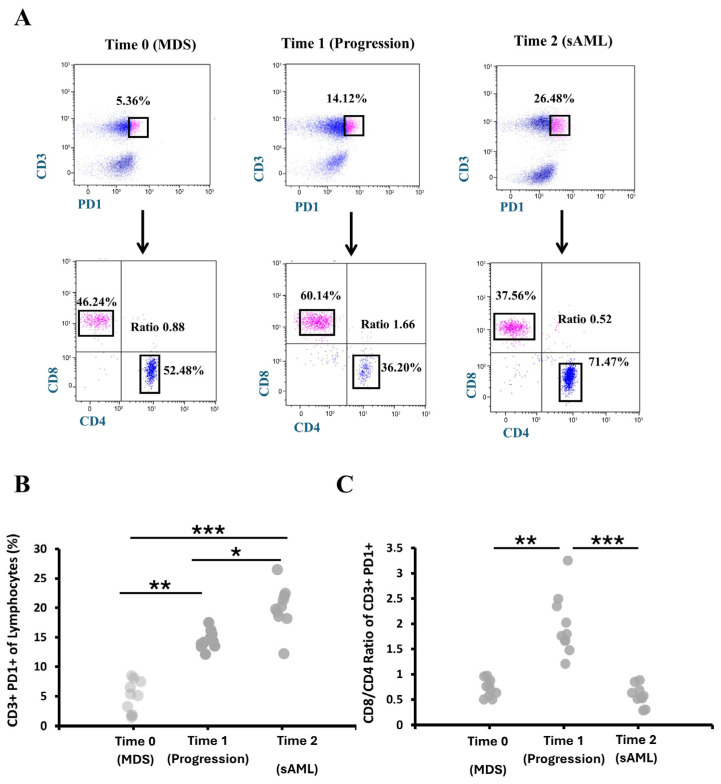
Immunophenotypic characterization of the CD8^+^/CD4^+^ ratio (PERLS) within CD3^+^ PD-1^+^ T cells in bone marrow across study time points. (**A**) Representative percentages of CD3^+^PD-1^+^ T cells (pink dots) and the CD8^+^/CD4^+^ (PERLS) ratio gated within the CD3^+^PD-1^+^ population from a single patient at Time 0 (MDS), Time 1 (disease progression), and Time 2 (secondary AML, sAML). (**B**) Percentages of CD3^+^PD-1^+^ T cells from all patients (n = 10) at the same time points. (**C**) CD8^+^/CD4^+^ (PERLS) ratio within CD3^+^PD-1^+^ T cells in the same cohort of 10 patients monitored at Time 0, Time 1, and Time 2. Statistical significance is indicated by * *p* < 0.05, ** *p* < 0.01, and *** *p* < 0.001.

To further characterize immunophenotypic changes associated with disease progression, we analyzed the proportions of CD8^+^PD-1^+^ and CD4^+^PD-1^+^ T cells across longitudinal time points. Representative flow cytometry plots from a single patient are shown in Figure 4A, depicting the CD3^+^PD-1^+^ population at Time 0 (MDS), Time 1 (early progression), and Time 2 (sAML) and the distribution of CD8^+^PD-1^+^ and CD4^+^PD-1^+^ cells, along with the corresponding CD8^+^/CD4^+^ ratio (PERLS). Quantitative analysis confirmed a progressive increase in CD3^+^PD-1^+^ T cells across disease stages (Figure 4B), with mean percentages (±SD) of 4.92% ± 3.5% at Time 0, 14.53% ± 2.68% at Time 1, and 20.05% ± 7.16% at Time 2.

From Time 0 to Time 1, PD-1^+^ CD8^+^ T cells increased while PD-1^+^ CD4^+^ T cells decreased, reflecting early immune activation. Following therapy modification at Time 1, this pattern reversed at Time 2, with PD-1^+^ CD8^+^ T cells decreasing and PD-1^+^ CD4^+^ T cells increasing in each patient (Supplementary Table S3). These opposing dynamics drove corresponding changes in the CD8^+^/CD4^+^ ratio (PERLS) within the CD3^+^PD-1^+^ compartment, which increased from 0.73 ± 0.48 at Time 0 to 1.97 ± 0.26 at Time 1 and subsequently decreased to 0.58 ± 1.16 at Time 2 (Figure 4C; individual values in Supplementary Table S3).

Additionally, at diagnosis (Time 0), PERLS values (CD8^+^/CD4^+^ ratio within the CD3^+^PD-1^+^ compartment) were higher in IPSS-R low-risk patients compared with intermediate/high-risk patients (Supplementary Table S3), consistent with more pronounced early immune activation. Median PERLS values remained higher in low-risk patients at progression (Time 1) (Time 0: 0.80 vs. 0.69; Time 1: 1.45 vs. 1.05), although these differences did not reach statistical significance (Mann–Whitney U test, *p* ≈ 0.69 and *p* ≈ 0.58, respectively).

In the longitudinal subset of patients with paired samples at diagnosis and progression (n = 10), changes in PERLS (ΔPERLS) and CD34^+^ blast percentages (ΔCD34) were calculated for each patient. Patients were stratified into low-risk and intermediate/high-risk groups according to their IPSS-R scores, as reported in Table 1, and Spearman rank correlation analyses were performed within each subgroup. No significant correlation was observed in the low-risk group (Spearman’s ρ ≈ −0.10), while a weak positive trend was noted in the intermediate/high-risk group (Spearman’s ρ ≈ 0.30), likely reflecting the limited sample size and biological heterogeneity.

## 4. Discussion

The progression from MDS to AML represents a biologically complex and clinically heterogeneous process, and growing evidence suggests that the bone marrow immune microenvironment plays a central role in shaping disease evolution [36,37], consistent with chronic immune dysregulation as a key feature in MDS pathogenesis [38]. Our study contributes to this understanding by providing a longitudinal immunophenotypic assessment in a well-characterized MDS cohort, highlighting early and dynamic alterations in T-cell dysfunction that precede overt leukemic transformation.

Our study design followed contemporary diagnostic standards. Patients were risk-stratified using IPSS-R, which incorporates bone marrow blast percentage, peripheral blood counts, and cytogenetic abnormalities. Limited molecular profiling of selected target genes was performed. However, comprehensive molecular studies were not included, as the study preceded the widespread implementation of IPSS-M in 2022, which integrates somatic mutation data with clinical and cytogenetic parameters to refine prognostic assessment in MDS [39]. However, in our cohort, available cytogenetic data showed that most MDS patients had normal or only subtly abnormal karyotypes, yet all fulfilled established morphologic and clinical diagnostic criteria (Supplementary Table S1). These observations align with previous reports showing that MDS can occur in patients with normal cytogenetics or without detectable somatic mutations and that molecular profiling, while valuable for prognostic stratification, is not independently diagnostic [40,41,42]. In all AML cases, cytogenetic and molecular analyses were performed as available, with molecular testing limited to the hospital-provided gene panel (Table 1). Accordingly, AML cases were assigned FAB subtypes (M0–M7), reflecting the morphology-based framework that continues to be clinically supported for subclassification, particularly in contexts where comprehensive molecular data are limited, as highlighted in a recent review [43].

Having stratified patients, we sought to understand how immune dysfunction unfolds across these risk groups, knowing that immune dysregulation differs across low-, intermediate-, and high-risk MDS [24]. Notably, PD-1 and CD57 expression on bone marrow CD8^+^ T cells peaked early at leukemic transformation (Time 1; Figure 3), consistent with progressive T-cell exhaustion in myeloid neoplasms [44]. This upregulation reflects an increasingly immunosuppressive microenvironment that facilitates clonal expansion, correlates with impaired cytotoxicity and disease aggressiveness in AML, and is generally more pronounced than in MDS [14,45]. Following this peak at transformation, exhaustion marker expression declined slightly at Time 2 (sAML) and subsequently stabilized after therapy initiation at Time 1 (progression) (Figure 3). These dynamics align with reports that chemotherapy and hypomethylating agents modulate T-cell exhaustion and PD-1/PD-L1 expression in myeloid neoplasms, supporting the view that immune dysfunction is dynamic rather than terminal [46,47].

Our longitudinal analysis of PD-1^+^ CD8^+^ and CD4^+^ T-cell dynamics, along with the PERLs CD8/CD4 ratio, highlights that immune perturbations occur early and dynamically during MDS progression. The transient increase in CD8^+^PD-1^+^ cells and concomitant decrease in CD4^+^PD-1^+^ cells at early progression suggest shifts toward a more cytotoxic yet potentially dysfunctional T-cell compartment, preceding overt leukemic transformation [45]. The subsequent reversal after therapy, including modulation of the PERLs ratio, underscores the plasticity of immune remodeling and the influence of treatment on T-cell phenotypes [48]. These observations align with prior reports of immune dysregulation as an early feature in MDS, including altered T-cell activation/exhaustion profiles and dynamic CD8/CD4 ratios [38,48]. In particular, the directional changes in the PERLs ratio support the notion that CD8/CD4 imbalance may serve as a biomarker for impending leukemic transformation, complementing traditional risk assessment.

Notably, although individual patient-level correlation between ΔPERLS and ΔCD34^+^ blasts was not statistically significant, likely due to the limited cohort size and biological heterogeneity, the stratification of patients by IPSS-R in low- and intermediate/high-risk groups revealed consistent trends. Low-risk MDS patients exhibited higher median PERLS values both at diagnosis and progression compared with intermediate/high-risk patients (Supplementary Table S3). Within the longitudinal subset, Spearman correlation analyses, between changes in PERLS (ΔPERLS) and CD34^+^ blasts (ΔCD34), showed no significant association in the low-risk group (ρ ≈ −0.10) and a weak positive trend in the intermediate/high-risk group (ρ ≈ 0.30). These results suggest that, although PERLS and blast expansion are not tightly coupled at the individual level, the directional trends, higher immune activation in low-risk and weaker activation in higher-risk patients, highlight the biological relevance of T-cell remodeling during MDS progression. Integrating these longitudinal immune measurements into standard risk stratification may help identify patients at higher risk of progression earlier and inform immune-targeted therapeutic strategies [27,49].

Overall, this study provides preliminary evidence that longitudinal immunologic monitoring, particularly of PD-1^+^ cells and the PERLs CD8/CD4 ratio, may assist in identifying MDS patients at elevated risk for progression. These findings support the integration of immune biomarkers into precision medicine-based, risk-adapted management strategies for MDS, highlighting that immune dysregulation can serve as a therapeutic target [26]. Further studies with larger, prospectively followed cohorts will be critical to validate these observations and refine immunologic tools for early detection of leukemic transformation.

## Figures and Tables

**Table 1:** Clinical, morphological, cytogenetic, molecular characteristics and therapy of secondary AML patients. (IPSS-R score: 1 = low, 2 = low, 3 = intermediate, 4 = high, 5 = high).

A/A	Age	MDS-Type	IPSS-R	AML-Type	Gene Mutation	Karyotype	Therapy (MDS → sAML)
1	57	MDS-SLD	1	M1	NPM1	46, XY	Azacitidine (low) → Cytarabine
2	72	MDS-EB-1	5	M0	Not detected	Complex *	Azacitidine (low) → Azacitidine (high)
3	68	MDS-U	1	M5	FLT3	46, XX	Supportive care → Cytarabine
4	75	MDS-SLD	1	M2	Not detected	46, XY del(20)(q12)	Azacitidine (low) → Azacitidine (high)
5	79	MDS-SLD	1	M0	FLT3	45, X (−Y)	Azacitidine (low) → Azacitidine (high)
6	47	MDS-MLD	2	M0	Not detected	Trisomy 8	Azacitidine (low) → Cytarabine
7	44	MDS-EB-2	4	M4	Not detected	46, XX	Azacitidine (high) → Cytarabine
8	76	MDS-EB-2	3	M4	FLT3	46, XY	Azacitidine(low) → Cytarabine
9	76	MDS-EB-1	3	M1	RUNX1	46, XX	Azacitidine (low) → Azacitidine (high)
10	58	MDS-EB-1	3	M4	NPM1	46, XX t(3;4)	Azacitidine (low) → Cytarabine

* The full karyotype notation (46,XY,del(7)(q31q33),-8,add(11)(p11.2),-12,+15, 17,add(19)(p13.3),+22,+4mar[cp7]/46,XX [13].

## Data Availability

The data supporting the findings of this study are available from the corresponding author upon reasonable request.

## Supplementary Materials

The following supporting information can be downloaded at: https://www.mdpi.com/article/10.3390/hematolrep18020029/s1, Supplementary Figure S1. Representative Flow Cytometric Assessment of Blast Population in Bone Marrow across Study Time Points.; Supplementary Table S1. Clinical, morphological, cytogenetic, molecular characteristics and therapy of MDS patients.; Supplementary Table S2. Percentages of CD34^+^ blasts in bone marrow across study time points.; Supplementary Table S3. Percentages of CD8^+^ and CD4^+^ cells gated in CD3^+^PD1^+^ T cells in bone marrow across study time points.

## Abbreviations

The following abbreviations are used in this manuscript:

MDS	Myelodysplastic syndromes
AML	Acute Myeloid Leukemia
LSC	Leukemic Stem Cells
RA	Refractory Anemia
RT	Refractory Thrombocytopenia
RN	Refractory Neutropenia
RARS	Refractory Anemia with Ring Sideroblasts
RCMD	Refractory Cytopenia with Multilineage Dysplasia
RAEB I	Refractory Anemia with Excess Blasts I
RAEB II	Refractory Anemia with Excess Blasts II
MSD-U	Myelodysplastic Syndrome Unclassifiable
IPSS-R	Revised International Prognostic Scoring System for MDS
IPSS-M	Molecular International Prognostic Scoring System for MDS
FAB	French–American–British Classification of AML
PD-1	Programmed Cell Death Protein 1
PD-L1	Programmed Death-Ligand 1
PERLS	CD8^+^PD-1^+^/CD4^+^PD-1^+^ Ratio
CTL	Cytotoxic T Lymphocyte
