# Supplementary material for: The Prognostic Value of the CD8+PD-1+/CD4+PD-1+ (PERLS) Ratio for Leukemic Transformation in MDS
Source: Hematol Rep. 2026 Apr 15;18(2):29. doi: 10.3390/hematolrep18020029 (PMC13116684; doi:10.3390/hematolrep18020029)
Supplement: Supplementary file 1 [file hematolrep-18-00029-s001.zip › hematolrep-4137849-supplementary.pdf]

hematology reports

Article

The prognostic value of the $CD8^+PD\text{-}1^+/CD4^+PD\text{-}1^+$ (PERLS) ratio for leukemic transformation in MDS

Panagiotis Panagiotidis [1,2], Emmanuel Karavanis [1], Konstantinos Neanidis [1], Eleftherios Panteris [2,3] and Maria Moysidou [2,*]

1 Oncology/Hematology Clinic, 424 General Military Hospital (424 GMHT), 56429 Thessaloniki, Greece;

2 Biomedical Sciences Unit, Faculty of Health Sciences, Metropolitan College, Campus of Thessaloniki, 54626 Thessaloniki, Greece; epanteris@mitropolitiko.edu.gr

3 Neonatology/Neonatal Intensive Care Unit (NICU), University General Hospital of Heraklion, University of Crete, 71003 Heraklion, Greece

* Correspondence: mmoysidou1@mitropolitiko.edu.gr

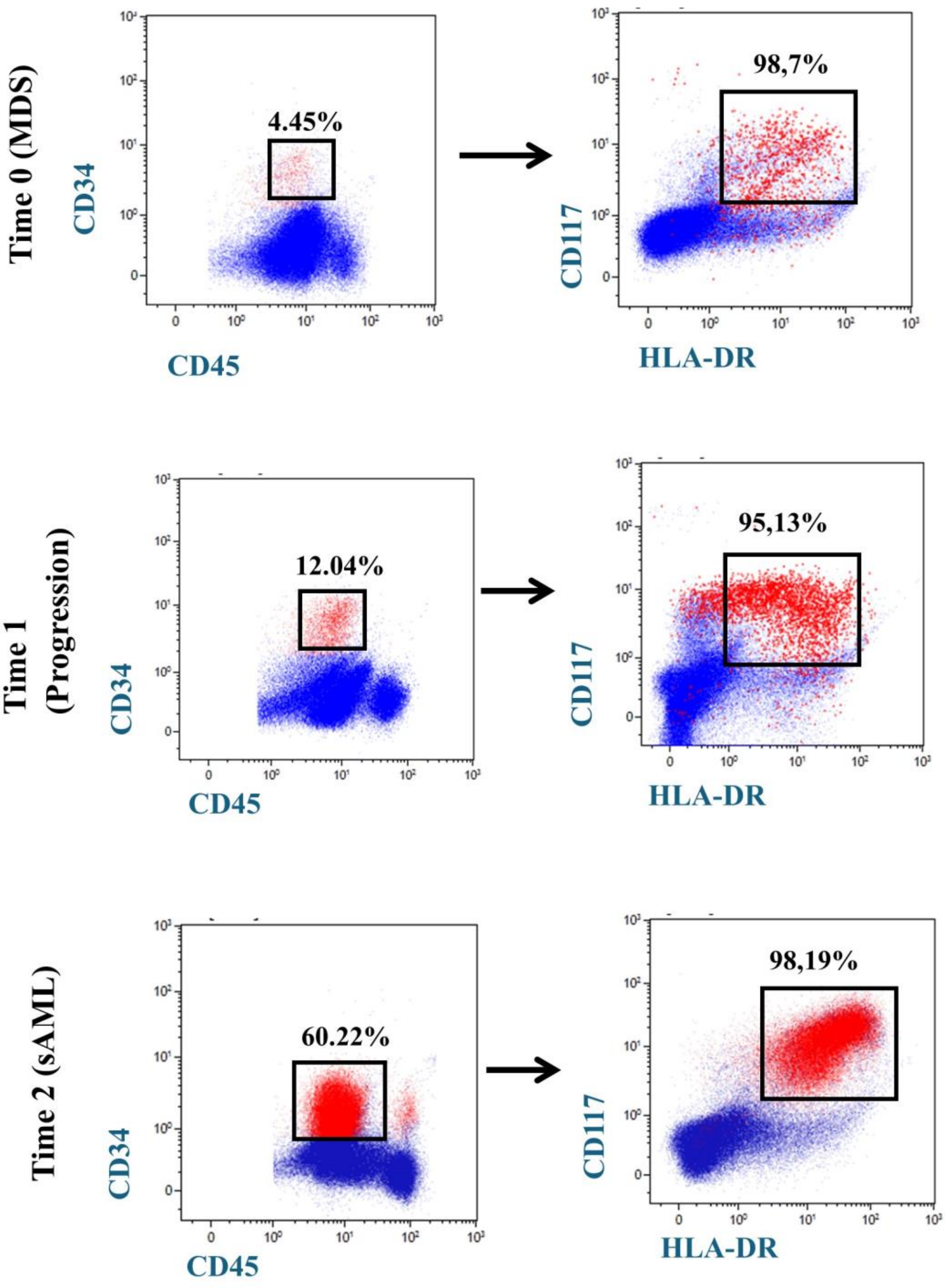

Supplementary Figure S1. Representative Flow Cytometric Assessment of Blast Population in Bone Marrow across Study Time Points. Representative plots from a single patient at three consecutive time points: Time 0 (MDS), Time 1 (disease progression), and Time 2 (sAML). $CD34^{+}$ blasts were gated within $CD45^{+}$ bone marrow cells. Percentages of $CD34^{+}$ blasts co-expressing $HLA\text{-}DR^{+}$ (FITC) and $CD117^{+}$ (PE) were quantified at each time point.

Supplementary Table S1. Clinical, morphological, cytogenetic, molecular characteristics and therapy of MDS patients.

A/A	Age	MDS-Type	IPSS-R	Karyotype	Gene Mutation	Therapy MDS
1	59	MDS-MLD	2	46, XX	Not detected	Supportive
2	35	MDS-EB-1	3	46, XY	Not detected	Azacitidine
3	74	MDS-U	1	46, XY	Not detected	Azacitidine
4	78	MDS/MPN	1	-	JAK2 (V617F)	Supportive
5	66	MDS-EB-1	5	Complex	Not detected	Azacitidine
6	53	MDS-EB-2	5	46, XX del(12)	Not detected	Azacitidine
7	81	MDS-MLD	2	45, X (-Y)	Not detected	Supportive
8	74	MDS-SLD	1	-	-	Supportive
9	81	MDS/MPN	1	46, XX	JAK2 (V617F)	Supportive
10	67	MDS-SLD	1	46, XY	-	Supportive
11	75	MDS-SLD	1	-	-	Supportive
12	59	RAEB II	4	46, XY	Not detected	Azacitidine
13	60	MDS-SLD	1	-	-	Supportive

(IPSS-R score: 1 = low, 2 = low, 3 = intermediate, 4 = high, 5 = high)

Supplementary Table S2. Percentages of $CD34^+$ blasts in bone marrow across study time points. The table shows the fraction of $CD34^+$ blasts within $CD45^+$ bone marrow cells for each patient at three consecutive time points: Time 0 (MDS), Time 1 (disease progression), and Time 2 (secondary AML).

A/A	AGE	IPSS-R	$CD34^+$ blasts (%) Time 0 (MDS)	$CD34^+$ blasts (%) Time 1 (Progression)	$CD34^+$ blasts (%) Time 2 (sAML)
1	57	1	1.47	14.30	50.75
2	72	5	6.68	16.39	25.78
3	68	1	4.00	8.93	21.44
4	75	1	1.73	8.52	70.59
5	79	1	2.63	12.04	31.68
6	47	2	3.95	10.84	60.22
7	44	4	5.43	24.12	70.44
8	76	3	4.45	31.04	70.12
9	76	3	3.79	18.06	34.19
10	58	3	5.12	23.05	75.22

Supplementary Table S3. Percentages of $CD8^+$ and $CD4^+$ cells gated in $CD3^+PD1^+$ T cells in bone marrow across study time points. The table shows the fraction of $CD8^+$ and $CD4^+$ cells gated in $CD3^+PD1^+$ **T** Cells in bone marrow for each patient at three consecutive time points: Time 0 (MDS), Time 1 (disease progression), and Time 2 (sAML).

A/A	AGE	IPSS-R	$CD8^+$ / $CD4^+$ (% of $CD3^+PD\text{-}1^+$) Time 0 (MDS)	$CD8^+$ / $CD4^+$ (% of $CD3^+PD\text{-}1^+$) Time 1 (Progression)	$CD8^+$ / $CD4^+$ (% of $CD3^+PD\text{-}1^+$) Time 2 (sAML)
1	57	1	46,14/47,45	53,22/44,07	44,58/50,44
2	72	5	35,69/56,27	62,5/37,43	45,98/54,02
3	68	1	46,24/52,48	63,79/36,21	34,11/59,35
4	75	1	39,22/49,22	70,16/29,84	22,91/75,63
5	79	1	29,88/59,14	75,7823,31	20,34/70,20
6	47	2	34,79/60,18	66,93/33,07	33,94/65,42
7	44	4	33,76/66,52	59,42/40,21	40,11/58,14
8	76	3	40,35/58,24	63,18/35,13	37,56/71,47
9	76	3	45,22/47,11	60,14/36,20	42,14/66,34
10	58	3	39,27/51,17	70,68/28,33	31,28/58,13

Disclaimer/Publisher's Note: The statements, opinions and data contained in all publications are solely those of the individual author(s) and contributor(s) and not of MDPI and/or the editor(s). MDPI and/or the editor(s) disclaim responsibility for any injury to people or property resulting from any ideas, methods, instructions or products referred to in the content.

https://doi.org/10.3390/hematolrep18020029
